# Transfer of *miR-100* and *miR-125b* increases 3D growth and invasiveness in recipient cancer cells

**DOI:** 10.20517/evcna.2024.43

**Published:** 2024-07-29

**Authors:** Hannah M. Nelson, Shimian Qu, Liyu Huang, Muhammad Shameer, Kevin C. Corn, Sydney N. Chapman, Nicole L. Luthcke, Sara A. Schuster, Tellie D. Stamaris, Lauren A. Turnbull, Lucas L. Guy, Xiao Liu, Danielle L. Michell, Elizabeth M. Semler, Kasey C. Vickers, Qi Liu, Jeffrey L. Franklin, Alissa M. Weaver, Marjan Rafat, Robert J. Coffey, James G. Patton

**Affiliations:** ^1^Laboratory of James G. Patton, Department of Biological Sciences, Vanderbilt University, Nashville, TN 37235, USA.; ^2^Laboratory of Marjan Rafat, Department of Biomedical Engineering, Vanderbilt University, Nashville, TN 37232, USA.; ^3^Laboratory of Qi Liu, Department of Biostatistics, Vanderbilt University Medical Center, Nashville, TN 37232, USA.; ^4^Laboratory of Kasey C. Vickers, Department of Molecular Physiology and Biophysics, Vanderbilt University Medical Center, Nashville, TN 37232, USA.; ^5^Department of Cell and Developmental Biology, Vanderbilt University Medical Center, Nashville, TN 37235, USA.; ^6^Laboratory of Alissa M. Weaver, Department of Cell and Developmental Biology, Vanderbilt University School of Medicine, Nashville, TN 37235, USA.; ^7^Laboratory of Robert J. Coffey, Department of Medicine, Division of Gastroenterology, Hepatology and Nutrition, Vanderbilt University Medical Center, Nashville, TN 37232, USA.

**Keywords:** miRNA, *miR-100*, * miR-125b*, colorectal cancer, cingulin, invasiveness, tight junctions

## Abstract

**Aim:**

Extracellular communication via the transfer of vesicles and nanoparticles is now recognized to play an important role in tumor microenvironment interactions. Cancer cells upregulate and secrete abundant levels of *miR-100* and *miR-125b* that can alter gene expression in donor and recipient cells. In this study, we sought to identify targets of *miR-100* and *miR-125b* and conclusively demonstrate that microRNAs (miRNAs) can be functionally transferred from donor to recipient cells.

**Methods:**

To identify targets of *miR-100* and *miR-125b*, we used bioinformatic approaches comparing multiple colorectal cancer (CRC) cell lines, including knockout lines lacking one or both of these miRNAs. We also used spheroid and 3D growth conditions in collagen to test colony growth and invasiveness. We also used Transwell co-culture systems to demonstrate functional miRNA transfer.

**Results:**

From an initial list of 96 potential mRNA targets, we identified and tested 15 targets, with 8 showing significant downregulation in the presence of *miR-100* and *miR-125b*. Among these, cingulin (CGN) and protein tyrosine phosphatase receptor type-R (PTPRR) are downregulated in multiple cancers, consistent with regulation by increased levels of *miR-100* and *miR-125b.* We also show that increased cellular levels of *miR-100* and *miR-125b* enhance 3D growth and invasiveness in CRC and glioblastoma cell lines. Lastly, we demonstrate that extracellular transfer of *miR-100* and *miR-125b* can silence both reporter and endogenous mRNA targets in recipient cells and also increase the invasiveness of recipient spheroid colonies when grown under 3D conditions in type I collagen.

**Conclusion:**

*miR-100* and *miR-125b* target multiple mRNAs that can regulate 3D cell-autonomous growth and invasiveness. By extracellular transfer, *miR-100* and *miR-125b* can also increase colony growth and invasiveness in recipient cells through non-cell-autonomous mechanisms.

## INTRODUCTION

MicroRNAs (miRNAs) are small (22-23nt) RNAs that base pair with target mRNAs to regulate gene expression by decreasing translation and promoting RNA degradation^[1,2]^. In cancer, miRNA expression patterns are frequently altered, contributing to all aspects of tumor progression from proliferation to metastasis^[3]^. We and others have focused on the role of *miR-100* and *miR-125b* in cancer^[4-9]^. These miRNAs are encoded in the third intron of *MIR100HG*, a 3kb lncRNA that is transcribed from the large 330 kb *MIR100HG* locus^[10]^. Increased expression of *miR-100*, *miR-125b*, and *MIR100HG* can promote cetuximab (anti-EGFR) resistance in colorectal cancer (CRC) cells, drive the progression of pancreatic cancer, and promote the epithelial to mesenchymal transition (EMT)^[7,8,11]^. These effects are driven in part by regulating the expression of target mRNAs including mTOR^[4]^, negative regulators of Wnt signaling^[8]^, cystic fibrosis transmembrane regulator (CFTR)^[9]^, cingulin (CGN)^[9]^, and checkpoint kinases (CHK)^[11]^. Beyond these mRNAs, TargetScan predicts a large number of additional potential targets for *miR-100* (59 targets) and *miR-125b* (931 targets) (https://www.targetscan.org/vert_80/). Base pairing between miRNAs and target 3’-UTRs is typically imperfect, with canonical targets displaying extensive pairing between nucleotides 2-8 of the miRNA (the “seed” sequence) and variable complementarity throughout the rest of the miRNA^[12]^. However, even within the seed sequence, the extent of base pairing between miRNAs and their mRNA targets is highly variable, making prediction of mRNA targets a challenge^[13,14]^. Indeed, all 5 of the negative regulators of Wnt (DKK1, DKK3, ZNRF3, RNF43, and APC2) that we previously found to be targeted by *miR-100* and *miR-125b* are noncanonical targets, raising the question as to whether additional targets might also contribute to cetuximab resistance and altered 2D and 3D growth.

Extracellular vesicles (EVs) and nanoparticles participate in cell-cell communication through the transfer of RNA, protein, and lipids^[15,16]^. Among RNA cargo, the transfer of miRNA is the most well-characterized due to their small size and the ability to experimentally monitor the silencing of target mRNAs in recipient cells using reporter constructs^[17]^. We previously showed that *miR-100* and *miR-125b* are secreted from CRC cells, with Transwell co-culture experiments supporting the extracellular transfer of these miRNAs to recipient cells^[4]^. However, since the recipient cells in those transfer experiments expressed endogenous *miR-100* and *miR-125b*, it remained a possibility that the observed silencing could be due to unexpected activation of the endogenous genes or other indirect effects^[18]^.

To identify additional targets of *miR-100* and *miR-125b* and to definitively demonstrate extracellular transfer, we created cell lines with CRISPR/Cas9-mediated knockouts of *miR-100*, *miR-125b*, or both. Through extensive bioinformatic analyses of RNAseq data, we identified and tested 15 candidate targets for *miR-100* and *miR-125b*, with the two most statistically significant being CGN and protein tyrosine phosphatase receptor type R (PTPRR). We also show that *miR-100* and *miR-125b* contribute to enhanced 3D growth and invasiveness of CRC and glioblastoma cells. Importantly, we demonstrate the functional transfer of *miR-100* and *miR-125b* from donor to recipient cells, leading to the silencing of both reporter constructs and endogenous genes, as well as increased invasiveness of spheroid cultures grown in type I collagen.

## MATERIALS AND METHODS

### Cell lines

DLD-1 cells are a heterozygous KRAS colorectal cancer cell line that was originally isolated by Daniel L. Dexter. The Sasazuki lab later generated isogenic KRAS lines (DKO-1 and Dks-8) from the parental DLD-1 cells and generously provided them for our use. These cells are also available from ATCC (CCL-221) and from Horizon Discovery. The LN-18 glioblastoma line was generously provided by the King lab at Vanderbilt and is also available from ATCC (CRL-2610).

### Cell culture

The CRC cell line HCA-7 was plated in 3D culture of type I collagen to generate cystic colonies (CC) cells^[8]^. CC cells were then subjected to iterative selection in 2D (cetuximab resistant) and 3D (cetuximab sensitive) conditions until a resistant line (CC-CR) was generated that is capable of growth in 3D in the presence of cetuximab^[8]^. The LN-18 glioblastoma line (ATCC CRL-2610) was a kind gift from the lab of Dr. Michael King at Vanderbilt. All cell lines were confirmed to be free of mycoplasma contamination [Venor^TM^GeM Mycoplasma Detection Kit, polymerase chain reaction (PCR)-based, Sigma Aldrich Cat #MP0025]. Cells were grown in Dulbecco’s Modified Eagle’s Medium (DMEM, Corning) supplemented with 10% fetal bovine serum (FBS, Corning), 1% L-glutamine (200 nM, Gibco), 1% MEM nonessential amino acids (Sigma Aldrich), and 1% penicillin-streptomycin (10,000 U/mL, Gibco) (termed complete DMEM) in 5% CO_2_ at 37 °C. Cells were passaged a maximum of 8 times before discarding.

### Generation of knockout cell lines

We used CRISPR/Cas9 multiplex genome engineering^[19]^ to delete *miR-100*, *miR-125b*, or both. Guide RNAs (gRNAs) flanking either *miR-100* or *miR-125b* or both were designed using the Benchling CRISPR gRNA Design Tool (https://www.benchling.com/crispr) [Supplementary Figure 1 and Supplementary Table 1]. Oligonucleotides containing gRNA sequences were first cloned into sgRNA expression vectors (Addgene #53186-53189) and then multiple gRNA cassettes were cloned into lentiviral expression vectors [pLV hUbC-Cas9-T2A-green fluorescent protein (GFP); Addgene, #53190] using Golden Gate cloning technology. Lentiviral stocks were prepared in 293T cells and used to transduce CC-CR cells. After transduction, single GFP^+^ cells were sorted into 96 cell plates and expanded. PCR amplification of genomic DNA from clonal GFP^+^ cells was performed using primers flanking the gRNA target sequences to identify deletion clones.

### EV isolation

Cells were plated at 9.0 × 10^6^ cells in T-175 flasks (Corning), with each EV collection using at least 3 flasks. Cells were grown to 80% confluency and washed 3 times with 1X phosphate-buffered saline (PBS) before media were replaced with DMEM lacking FBS and grown for 48 h. Culture media were then collected and adherent cells were trypsinized and counted. Conditioned media were then centrifuged at increasing speeds and time: 1,000 × *g* for 10 min (room temperature), 2,000 × *g* for 20 min (4 °C), and 10,000 × *g* for 30 min (4 °C). Crude EV pellets were obtained after the conditioned media was centrifuged for 17 h at 100,000 × *g* (4 °C). EV pellets were then washed two times by resuspension in 1 mL of 1X Dulbecco’s phosphate-buffered saline (DPBS) (Corning) and centrifugation at 100,000 × *g* for 70 min. Final pellets were resuspended in 100 µL 1X DPBS. Under these conditions, the EV pellets consist of a heterogeneous mixture of EVs with some non-vesicular material^[20]^. This protocol was used to directly compare with earlier results examining the export of *miR-100* and *miR-125b* into CRC EVs^[4]^. EVs were quantified by protein concentration (Pierce BCA Protein Assay) and by Nanoparticle Tracking Analysis (NTA, ZetaView Nanoparticle Tracking Analysis Instrument).

### Quantitative reverse transcripiotn polymerase chain reaction

Total RNA from both whole cells and EVs was isolated using TRIzol (Life Technologies). Taqman small RNA assays (Life Technologies) were used to quantify miRNA levels. A total of 10 ng of RNA was utilized for each reverse transcription (RT) reaction; 0.67 µL of cDNA was used in each 10 µL quantitative polymerase chain reaction (qPCR) reaction. qPCR was completed in either 96-well or 384-well plates using Biorad CFX96 or CFX384 instruments. Fold changes were calculated as previously described^[4]^.

### Transfer assays

For Transwell co-culture assays, cells were plated in Transwell dishes with 0.4 µm filters (Corning, 3460), with 50,000 cells in the donor well and 300,000 cells in the recipient well. Transwell co-cultures were grown for five days, changing media every 72 h. Cells were washed 3 times with 2 mL of 1X PBS; RNA was then collected from either donor or recipient cells.

### AGO2 immunoprecipitation

For immunoprecipitation, anti-AGO2 (clone 11A9 antibody; MABE253, Sigma-Aldrich) or control IgG antibodies were used. Ten 150 cm dishes of either CC or CC-CR cells were grown to 95% confluency. Cells were then scraped in ice-cold PBS and centrifuged to collect cell pellets in ice-cold lysis buffer (20 mM Tris HCl pH 7.5, 150 mM KCl, 0.5% NP40, 2 mM EDTA, 1 mM NaF, 0.5 mM DTT). After centrifugation of insoluble cell debris, cell lysates were incubated with Dynabeads. Prior to cell lysis, Dynabeads^TM^ Protein G (ThermoFisher, 10003D) were conjugated with antibodies by incubating overnight with rotation at 4 °C and washed two times with ice-cold lysis buffer. Cell lysates were then added to the conjugated beads and incubated at 4 °C for 6 h. Beads were gently washed with fresh lysis buffer and proteins were eluted. For RNA isolation, beads were incubated with DNase and Proteinase K for 10 min at room temperature. Trizol was then added and incubated at room temperature for 10 min and RNA was isolated.

### Small RNA sequencing

RNA libraries were generated using the NEXTFlex Small RNA Library Preparation Kits v3 (Perkin) with the following modifications: (1) 3’- and 5’-adaptors were diluted 1:8; (2) 3’-adaptor ligations were performed overnight in multiple steps: 25 °C for 2 h, 20 °C for 4 h, and 16 °C overnight; (3) following cDNA synthesis and before barcoding PCR, clean up was performed using the No Size Selection protocol (Perkin); and (4) PCR amplification was 20 cycles. Following PCR amplification, individual libraries were size-selected (136-200 bp product) using Pippin Prep (Sage Sciences). Size-selected libraries were quantified using a Qubit Fluorometer. Libraries were checked for quality and sequenced using Illumina short-read technology. Libraries were pooled and paired-end sequencing (PE-150) (equimolar multiplexed libraries) was performed on the NovaSeq6000 platform using the VANTAGE core (Vanderbilt University). Demultiplexing and bioinformatic analyses were performed using the TIGER pipeline^[21]^. Briefly, Cutadapt (v1.16) was used to trim 3’ adaptors and all reads with < 16 nucleotides (nts) were removed^[22]^. Quality control on both raw reads and adaptor-trimmed reads was performed using FastQC (v0.11.9) (www.bioinformatics.babraham.ac.uk/projects/fastqc). The adaptor-trimmed reads were mapped to the hg19 genome, with additional rRNA and tRNA reference sequences using Bowtie1 (v1.1.2), allowing only one mismatch^[23]^.

### Total RNA sequencing

Bulk RNA sequencing libraries were prepared using Universal RNAseq kits (Tecan). Libraries were cleaned (Zymo), checked for quality using the Agilent bioanalyzer, quantified (Qubit), and pooled based on equimolar concentrations. Pooled libraries were sequenced using Illumina short-read technology based on PE150 on the NovaSeq6000 (Illumina). After sequencing, samples (libraries) were demultiplexed and analyzed. Briefly, adapter sequences were removed using Cutadapt v2.10)^[22]^. Quality control of both raw reads and adaptor-trimmed reads was performed using FastQC (v0.11.9). After adaptor trimming, reads were aligned to the Gencode GRCh38.p13 genome using STAR (v2.7.8a)^[24]^. FeatureCounts (v2.0.2)^[25]^ was used to count the number of reads mapped to each gene. Heatmap3^[26]^ was used for cluster analysis and visualization. Differential expression was analyzed by DESeq2 (v1.30.1)^[27]^. Significant differentially expressed genes were determined with fold change > 2 or < 0.5, and adjusted *P* values (padj) < 0.05. Genome Ontology and KEGG pathway over-representation analyses were performed using the WebGestaltR package (NULL)^[28]^. Gene set enrichment analysis (GSEA) was performed using GSEA package (v4.3.2)^[29]^ on database v2022.1.Hs.

### Gene expression analysis

The Gene Expression database of Normal and Tumor Tissues (GENT2) was used to compare levels of expression between 72 paired normal and tumor tissues utilizing the GPL570 platform. Statistical significance was determined using two-sample *t*-tests [GPL570 platform (HG-U133_Plus_2)].

### Transfection of luciferase plasmids

Cells were grown to approximately 50%-60% confluency with transfections performed using TransIT®-2020 (Mirus, MIR5404). The Renilla luciferase construct (pClneo-RL, plasmid #115366, Addgene) was transfected at 0.5 μg/mL and the firefly luciferase construct was transfected at 1μg/mL. DNA and TransIT-2020 were incubated for 30 min at room temperature in Opti-MEM (Gibco). Transfection complexes were diluted 1:10 in DMEM and added to cells.

### Luciferase reporter assays

3’-UTRs from mRNAs of interest were cloned using RT/PCR into the pMIR-REPORT^TM^ miRNA Expression Reporter Vector System (Firefly Luciferase) using NEBuilder HiFi DNA Assembly (New England BioLabs). PCR amplification primers are listed in Supplementary Table 1. All plasmids were verified by sequencing (Genewiz). Transfected cells were cultured alone or in co-cultures using Transwells with 0.4 mm membranes for 72 h, followed by washing with 1X PBS. Luciferase levels were then detected using the Dual-Glo® Luciferase Assay System (Promega). Changes in firefly luciferase levels were normalized to Renilla luciferase levels and compared to luminescence from the empty (no 3’UTR) vector.

### Immunofluorescent staining and imaging of cells

For cell images, cells were plated on coverslips and grown for three days, after which coverslips were washed three times in 1X PBS and fixed in 4% paraformaldehyde for 30 min at room temperature. Samples were then washed and allowed to block for 1 h at room temperature in blocking buffer (1X PBS with 0.001% Triton-X and 0.03% donkey serum). Primary antibody incubation (CGN, ab244406, Abcam) was overnight at 4 °C, followed by three 1X PBS wash steps, and incubation with secondary antibodies (Cy3 anti-Rabbit antibody, Jackson ImmunoResearch and Alexa Fluor 488 conjugated phalloidin, ThermoFisher) for 2 h at room temperature. Samples were washed three times with 1X PBS and mounted on glass slides using Vectashield with 4’,6-diamidino-2-phenylindole (DAPI) and imaged using a Zeiss LSM 880 microscope (Vanderbilt University, CISR). All images were analyzed using ImageJ^[30]^.

For quantification of immunofluorescence in cells, cells on glass coverslips were fixed in neutral-buffered formalin and incubated in a 0.1% Triton X-100 solution (Sigma-Aldrich X100) for 10 min. Cells were blocked for 1 h at room temperature with 10% normal goat serum (Vector Labs S-1000) and 22.5 mg/mL glycine and then incubated overnight at 4 °C in a humidified chamber with anti-CGN (1:500) in 1% bovine serum albumin (Sigma A1470) and 0.1% TWEEN 20 (Sigma-Aldrich P1379). Coverslips were mounted onto slides using ProLong Glass Antifade Mountant with NucBlue (Invitrogen P36981) following secondary antibody incubation with goat anti-rabbit IgG AlexaFluor 594 (1:500, Invitrogen A-11012). A corresponding no primary antibody control was performed for all conditions to confirm specificity. Stained samples were imaged using a Leica DMi8 inverted microscope with Leica DFC9000GT sCMOS fluorescence digital camera. For fluorescence microscopy, a Lumencor mercury-free SOLA light engine was used for the illumination source. The microscope was fitted with DAPI, GFP, Texas Red (TXR), and Y5 filter cubes. Images (8 bit) were captured using LASX imaging software. Images of CGN were captured as Z-stacks with 0.5 μm spacing between slices. All images were analyzed using Fiji software^[31]^. The area of positive staining was set based on the no primary control images and thresholded accordingly. Z-stacks were merged using maximum intensity projections. For CGN analysis, the area of positive expression was normalized by nuclei number.

### Spheroid cultures

Cells (10,000) were plated in Nunclon Sphera low adherent plates (Thermo Scientific) and centrifuged at 1,000 × *g* for 10 min at room temperature, as previously described^[32]^ in complete DMEM. Cells were grown for three days before transfer into 2 mg/mL type I collagen (Advanced Biomatrix). Once collagen was solidified, complete DMEM was added and changed every two days. Colonies were imaged using Muvicyte Live Cell imager for five days. Images were processed and analyzed using ImageJ^[30]^.

### Overexpression of CGN

The CGN coding region was amplified by RT/PCR from RNA isolated from CC cells using primers as shown in Supplementary Table 1. PCR products were cloned into a Sleeping Beauty-based Tet-On vector (pSB829) using NEBuilder HiFi DNA assembly. The resulting construct was confirmed by sequencing. For transfections, 0.5 μg plasmid DNA (pCDNA3.1-SB100X) and 0.5 μg of overexpression vector were incubated with 2 μg of Lipofectamine 2000 (ThermoFisher) in Opti-MEM (Gibco) for 30 min at room temperature. Transfection complexes were diluted in 1:10 in complete DMEM and added to LN-18 cells and incubated for 24 h. Cells were then selected using 300 μg/mL of hygromycin. CGN expression was induced by adding 300 ng/mL of doxycycline (DOX).

### Protein collection and western blotting

Proteins were collected using 1X-RIPA buffer (Life Technologies) and quantified using BCA assays (ThermoFisher). 40 μg of the total protein was loaded onto pre-cast SDS gels (4%-20% Mini-PROTEAN TGX 50 μL pre-cast gels; BIO-RAD). Separated proteins were transferred to nylon membranes using the Trans-Blot Turbo Transfer System (BIO-RAD). Membranes were blocked with Intercept Blocking Buffer (IBB) (LI-COR) for 1 h at room temperature. Primary antibodies [CGN, Abcam and glyceraldehyde 3-phosphate dehydrogenase (GAPDH), ThermoFisher) were incubated overnight in IBB at 4 °C. Blots were washed three times in 1X TBS-T. Secondary antibodies (IRDye® 680 RD Donkey anti-Rabbit and IRDye® 800 CW Donkey anti-Mouse, LI-COR) were incubated for 1 h at room temperature in IBB. Blots were then washed in 1X TBS-T three times. Blots were imaged using the Odyssey XF (LI-COR) and quantified using ImageJ.

## RESULTS

**Figure 1:**
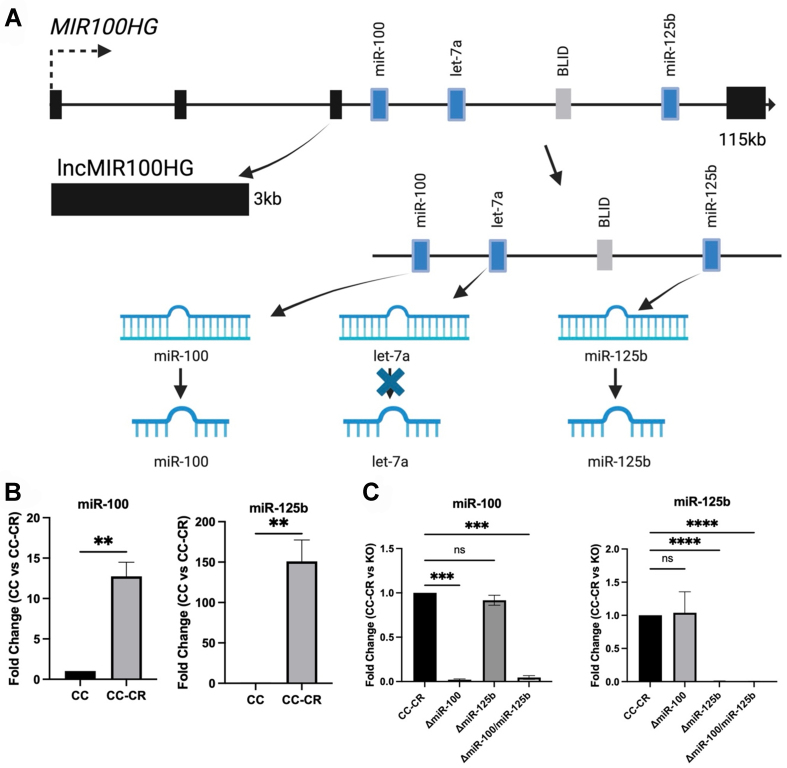
Expression levels of *miR-100* and *miR-125b* in CC, CC-CR and Δ*miR-100/miR-125b* cell lines. (A) One of multiple transcription start sites within the 330 kb *MIR100HG* locus encodes a 115 kb transcript that is spliced (black exons) to generate a 3 kb lncRNA (*MIR100HG*) with *miR-100*, *miR-125b*, *let-7a*, and the BLID protein encoded in the third intron. In CC-CR cells, *MIR100HG*, *miR-100*, and *miR-125b* are highly overexpressed, but *let-7a* and BLID are not^[8]^; (B) CC-CR cells are cetuximab-resistant cells derived from cetuximab-sensitive CC cells^[8]^. *miR-100* and *miR-125b* are dramatically overexpressed in CC-CR cells, as measured by RT-qPCR. Data (mean ± SEM, *n* = 3) were analyzed using Student’s *t* test; (C) RT-qPCR analysis of *miR-100* and *miR-125b* levels in CC-CR, Δ*miR-100*, Δ*miR-125b*, and Δ*miR-100/miR-125b* cell lines. Fold changes were determined using the ΔΔC(t) method. Data (mean ± SEM, *n* = 3) were analyzed using one-way ANOVA tests with ^**^ indicating *P* < 0.005, ^***^ indicating *P* < 0.001, and ^****^ indicating *P* < 0.0001. CC: Cystic colonies; RT-qPCR: reverse transcription-quantitative polymerase chain reaction; SEM: scanning electron microscopy; ANOVA: analysis of variance.

To facilitate the identification of mRNA targets for *miR-100* and *miR-125b* and to develop cell lines to test for functional miRNA transfer, we used CRISPR/Cas9 technology to generate deletions of these two miRNAs within the *MIR100HG* locus [Figure 1A]. A single lentiviral vector expressing multiple gRNAs, Cas9, and GFP was created and lentiviruses were transduced into cetuximab-resistant CC-CR cells^[8]^. The gRNAs were designed to base pair with regions flanking *miR-100*, *miR-125b*, or both [Supplementary Figure 1]. Targeting both *miR-100* and *miR-125b* also results in the deletion of *let-7a* and the BLID open reading frame, but previous work did not support a role for these genes in cetuximab resistance^[8]^. Individual GFP^+^ clones were sorted, targeted deletions were identified by PCR, and verified by sequencing. While CC-CR cells express high levels of *miR-100* and *miR-125b*, the parental, cetuximab-sensitive CC cell line expresses ~50-fold less of both miRNAs^[8]^ [Figure 1B]. As expected, the knockout lines showed undetectable levels of *miR-100* or *miR-125b* or both [Figure 1C].

### Identification of mRNA targets of *miR-100* and *miR-125b*

**Figure 2:**
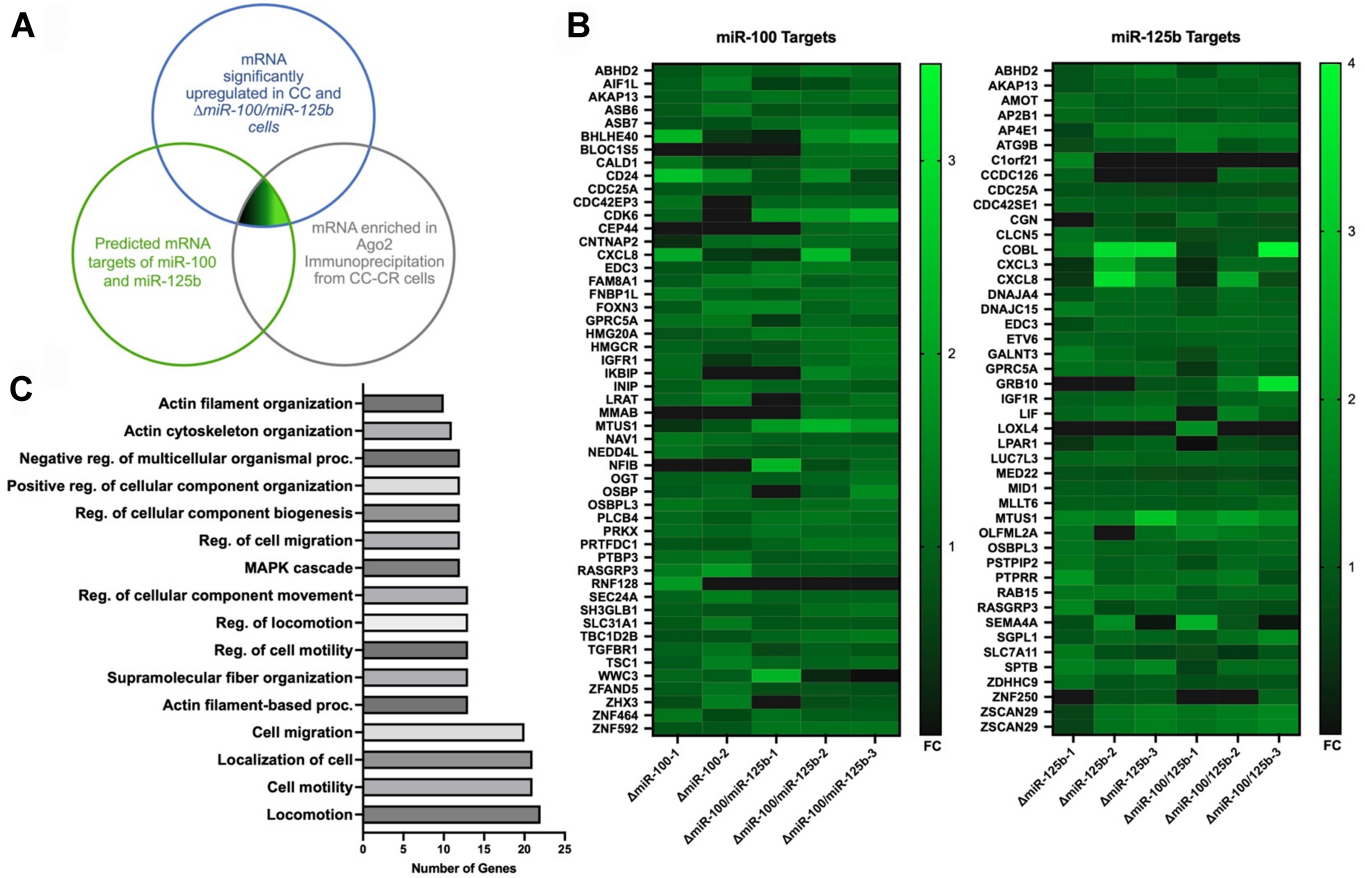
Identification of targets for *miR-100* and *miR-125b* in CRC cells. (A) Differentially expressed genes were identified by mining RNAseq data comparing CC, CC-CR, Δ*miR-100*, Δ*miR-125b*, Δ*miR-100/miR-125b* cells. Venn diagram showing the overlap between genes upregulated in CC and knockout cell lines, predicted mRNA targets of *miR-100* and *miR-125b* using three different algorithms, and association with AGO2 in CC-CR cells; (B) Heatmaps were generated from the differential RNAseq data to identify potential targets upregulated in CC cells (> 2 fold) and knockout cells (> 1.5 fold) compared to CC-CR cells; (C) Gene Ontology analysis of the targets of *miR-100* and *miR-125b* showed enrichment in genes associated with cell migration and motility. Figure was created using ShinyGO^[37]^. CRC: Colorectal cancer; CC: cystic colonies.

RNAseq was performed on CC, CC-CR, and knockout cell lines (Δ*miR-100*, Δ*miR-125b*, and Δ*miR-100/miR-125b*) and bioinformatic analyses were performed to compare gene expression patterns between the lines [Supplementary Table 2]. Potential mRNA targets for *miR-100* and *miR-125b* were identified using a three-step strategy [Figure 2A]. First, we identified upregulated mRNAs in both CC and Δ*miR-100/miR-125b* cells compared to CC-CR cells^[8]^. Second, we compiled a list of predicted targets for *miR-100* and *miR-125b* using three different prediction algorithms (TargetScan, TargetMiner, and miRDB; https://www.targetscan.org/vert_80/; https://www.isical.ac.in/~bioinfo_miu/targetminer20.htm; https://mirdb.org/). Third, we performed RNA immunoprecipitation of AGO2 followed by bulk and short RNA sequencing [Supplementary Tables 3 and 4]. AGO2 is a component of the RNA Induced Silencing Complex (RISC) in which miRNAs pair with their target mRNAs^[33-36]^. Immunoprecipitation of AGO2-containing complexes from CC-CR cells allowed for the enrichment of *miR-100* and *miR-125b* targets because these miRNAs are so highly expressed in CC-CR cells^[8]^. By comparing the three sets of data, we were able to identify targets of *miR-100* and *miR-125b* [Figure 2A], with 51 potential targets for *miR-100* and 45 potential targets for *miR-125b* [Figure 2B]. When we subjected all 96 potential targets to Gene Ontology analysis using ShinyGo^[37]^, we found significant enrichment for genes involved in cell migration, cell motility, actin filament-based processes, and MAPK signaling [Figure 2C and Supplementary Table 5].

### Silencing of mRNA targets of *miR-100* and *miR-125b*

**Figure 3:**
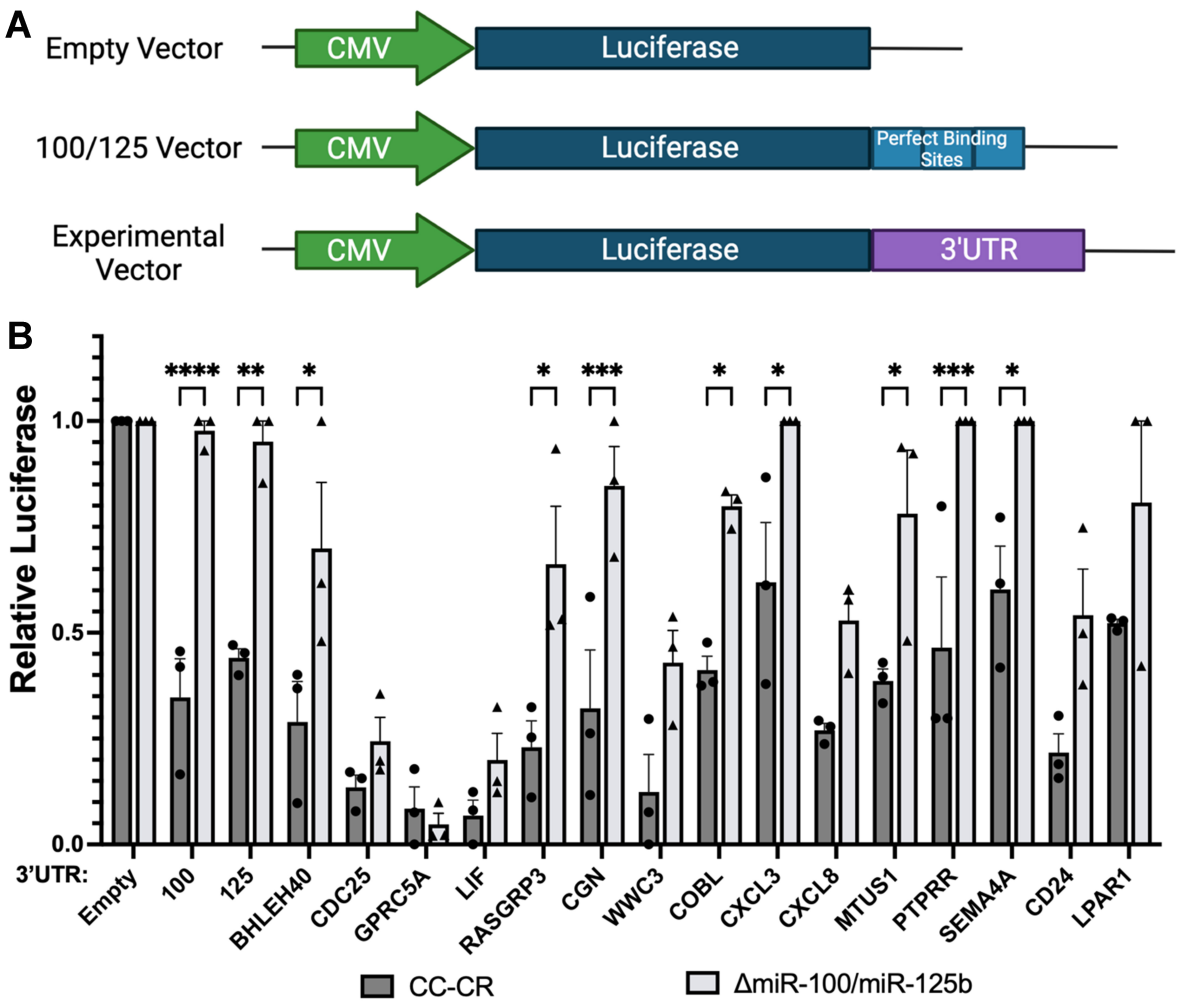
Silencing of reporter constructs containing target 3’-UTRs for *miR-100* and *miR-125b*. (A) Reporter constructs were generated in which the firefly luciferase open reading frame was fused to either plasmid 3’-UTR sequences (empty vector), 3’-UTR sequences containing three perfect binding sites for *miR-100 or miR-125b* (100/125 vector), or 3’-UTR sequences from candidate mRNA targets of *miR-100 or miR-125b* (experimental vector); (B) Quantification of relative luciferase levels after transfection of the indicated constructs into CC-CR cells or Δ*miR-100/miR-125b* cells. Relative firefly luciferase levels were calculated by normalizing to a co-transfected internal control vector expressing Renilla luciferase. Data (mean ± SEM, *n* = 3) were analyzed using two-way ANOVA tests, with ^*^ indicating *P* < 0.05, ^**^ indicating *P* < 0.01, ^***^ indicating *P* < 0.001, and ^****^ indicating *P* < 0.0001. SEM: Scanning electron microscopy; ANOVA: analysis of variance.

From the targets generated above, we examined miRNA:mRNA pairing [Supplementary Figure 2] and selected 15 candidate mRNAs to test for the ability of *miR-100* (3 candidates), *miR-125b* (7 candidates), or both (5 candidates) to silence luciferase reporter constructs. We designed three different categories of luciferase vectors in which the firefly luciferase open reading frame was fused to (1) no 3’-UTR (empty vector); (2) 3’-UTRs containing three perfect binding sites for *miR-100* or *miR-125b* (100/125 vector); or (3) 3’-UTRs from candidate targets (experimental vectors) [Figure 3A]. These vectors were transfected into either CC-CR or Δ*miR-100/miR-125b* cells and luciferase levels were determined. Bona fide targets of *miR-100* or *miR-125b* should show increased luciferase levels in Δ*miR-100/miR-125b* cells as compared to CC-CR cells. Of the 15 targets we tested, 14 showed increased luciferase levels in Δ*miR-100/miR-125b* cells, with 8 being statistically significant [Figure 3B]. Of the 8, CGN and PTPRR showed the most significant increase in luciferase activity when comparing Δ*miR-100/miR-125b* and CC-CR cells [Figure 3B]. The fact that the two most significant genes are targets of *miR-125b* is consistent with more dramatic overexpression of *miR-125b* in CC-CR cells as compared to *miR-100* [Figure 1B].

### CGN and PTPRR are downregulated in CRC

*miR-100* and *miR-125b* display elevated gene expression and target multiple mRNAs in CRC and other cancer types^[4,8,9,11,38-40]^. CC cells grown in 3D in type I collagen form hollow cysts with a central lumen lined by polarized cells, whereas CC-CR cells form solid, disorganized colonies, indicative of aggressive behavior^[8]^. We thus sought to test if decreased expression of CGN and PTPRR due to increased expression of *miR-100* and *miR-125b* might be observed in CRC and other cancers. We used the GENT2 database to compare the levels of CGN and PTPRR between normal and cancer tissues [Supplementary Figure 3A and B] and found that CGN is downregulated in 19/35 cancers including CRC (red brackets) while PTPRR is downregulated in 16/35 including CRC. There are likely additional mechanisms that can lead to decreased levels of CGN and PTPRR because not all cancers show increased expression of *miR-100*^[38]^ or *miR-125b*^[39]^, but the data are in agreement that decreased levels of CGN and PTPRR correlate with cancer.

**Figure 4:**
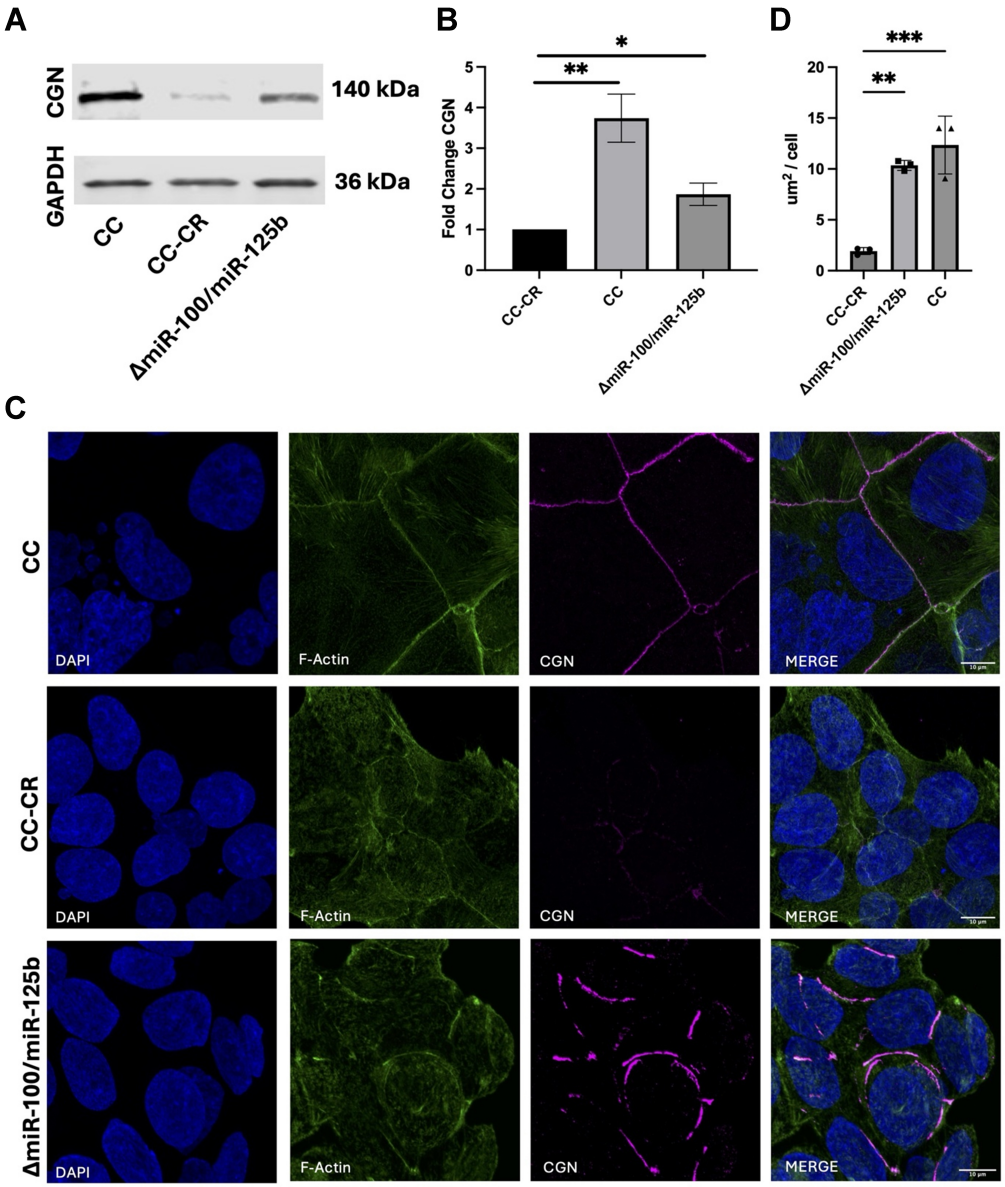
Decreased Expression of CGN in Cells Expressing High Levels of *miR-100* and *miR-125b*. (A and B) Western blots and quantitation of CGN levels in the indicated cell lines; (C) The indicated cells were fixed and immunostained with DAPI to stain nuclei (blue), Alexa Fluor 488-phalloidin to stain F-actin (green), or antibodies against CGN (magenta). Merged images are shown at the far right. Scale bar indicates 10 mm; (D) Quantification of CGN/cell for the indicated cell lines. For (B), data (mean ± SEM, *n* = 3) were analyzed using Student’s *t* test. For (C), data (mean ± SEM, *n* = 3) were analyzed using one-way ANOVA. For significance, ^*^ indicates *P* < 0.05, ^**^ indicates *P* < 0.01, ^***^ indicates *P* < 0.001. CGN: Cingulin; DAPI: 4’,6-diamidino-2-phenylindole; SEM: scanning electron microscopy; ANOVA: analysis of variance.

To test whether our CRC cell lines match the GENT2 data, we used antibodies to perform western blots and immunostaining of CGN protein levels in CC, CC-CR, and knockout cell lines. Even though we observed silencing of PTPRR reporter constructs [Figure 3], we did not detect reduced protein levels by western blots, so we decided to focus hereafter on CGN. CGN connects tight junctions to the cytoskeleton and localizes to apical junctions joining adjacent epithelial cells^[9,41]^. In cells that express low to undetectable levels of *miR-100 or miR-125b* (CC and Δ*miR-100/miR-125b* cells), CGN was readily detected by western blots [Figure 4A and B] and localized to cell-cell junctions by immunofluorescence [Figure 4C]. Quantification of fluorescent images demonstrated that cells with low or no *miR-100* and *miR-125b* had increased levels of CGN per cell compared to CC-CR cells with high expression of *miR-100* and *miR-125b* [Figure 4D]. Immunofluorescent signals were more readily visualized in CC cells compared to Δ*miR-100/miR-125b* cells, potentially due to the regulation of other factors by *miR-100 or miR-125b* that also seem to affect overall morphology, as indicated by differences in F-actin staining and changes in CGN localization. In cells expressing high levels of *miR-100* and *miR-125b* (CC-CR cells), CGN detection by both western blots and immunofluorescence was dramatically reduced [Figure 4]. These results are consistent with targeting of CGN by *miR-100* and *miR-125b*.

### *miR-100* and *miR-125b* contribute to enhanced 3D growth and invasiveness in CRC cells

**Figure 5:**
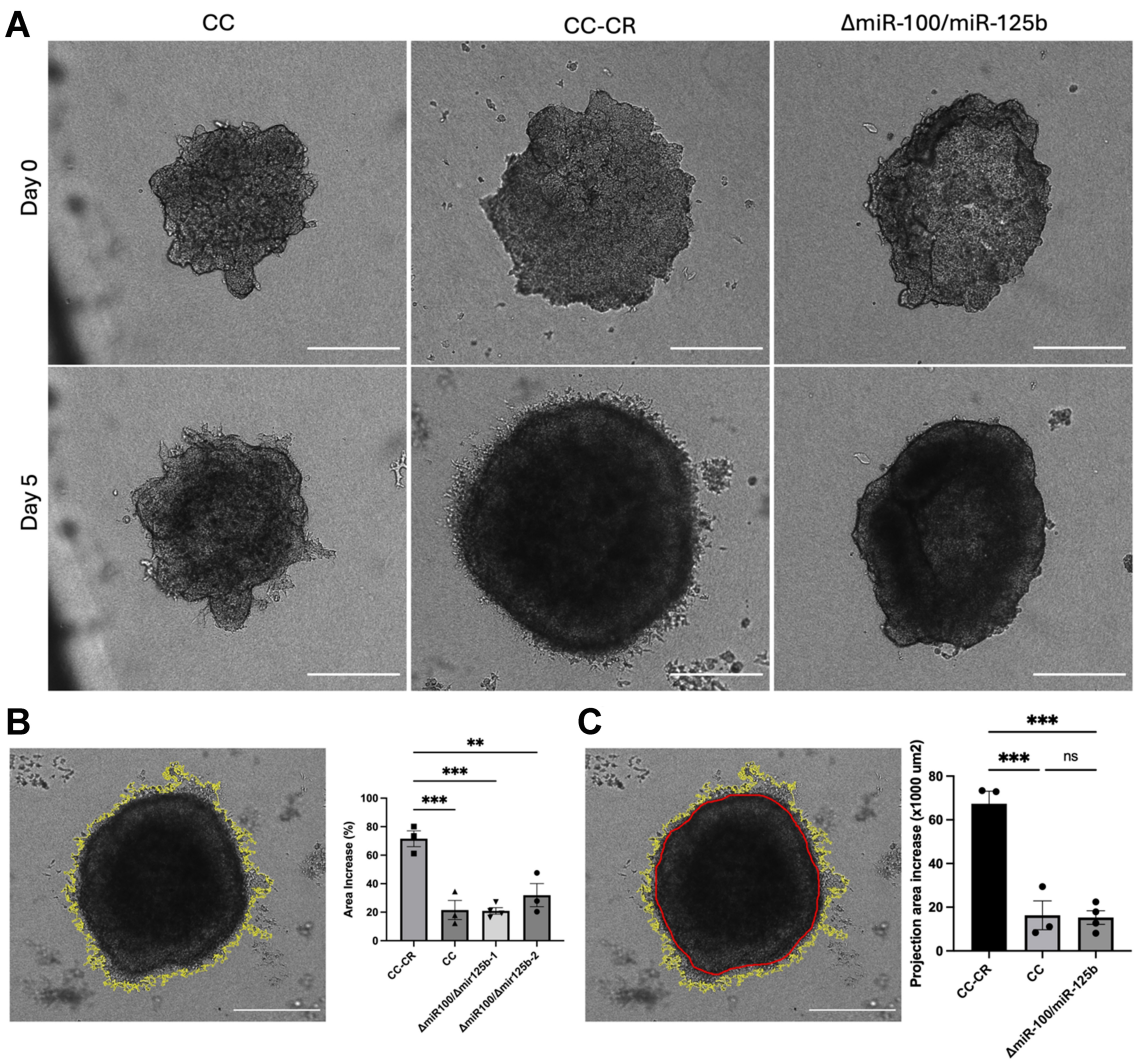
*miR-100* and *miR-125b* contribute to enhanced 3D growth and invasiveness. (A) The indicated cell types were plated in low adherent, spheroid cultures for 3 days before transfer to growth in type I collagen and live imaging over 5 days. Scale bar indicates 500 μm; (B) The increase in colony area was determined by measuring the surface area of spheroids from day 0 to day 5. Scale bar indicates 500 μm. Representative image is a CC-CR spheroid. Surface area tracing was performed using ImageJ, shown in yellow. Significance was analyzed using one-way ANOVA tests (*n* = 3); (C) Increases in area due to colony projections into the collagen (invasiveness) were calculated by measuring the total surface area including projections (yellow) and subtracting the surface area of the main spheroid (red). Scale bar indicates 500 μm. Representative image is a CC-CR spheroid. Significance was analyzed using one-way ANOVA tests, data (*n* = 3). Data represent mean ± SEM with ^**^ indicating *P* < 0.01 and ^***^ indicating *P* < 0.001; ns indicating not significant. ANOVA: Analysis of variance; SEM: scanning electron microscopy.

Gene Ontology analysis of targets of *miR-100* and *miR-125b* showed enrichment of genes associated with cell migration and motility [Figure 2C]. This is consistent with the downregulation of CGN, which leads to increased invasiveness, metastatic potential, and EMT^[9,42-44]^. To test the effect of *miR-100* and *miR-125b* and their targeting of CGN on 3D growth, we cultured CC, CC-CR, and Δ*miR-100/miR-125b* cells under spheroid growth conditions^[32]^ in low adherence plates for 3 days before transfer to growth in type I collagen. Under these conditions at both day 0 and day 5 in collagen, the morphology of Δ*miR-100/miR-125b* colonies was more similar to CC colonies than to colonies derived from parental CC-CR cells [Figure 5A and Supplementary Movies 1-3]. On day 5, we also observed significant differences in surface dynamics when comparing colonies derived from the three lines [Supplementary Movies 1-3]. Increased projections emanating from the edges of the colonies and protruding into collagen were observed with CC-CR cells, whereas far fewer projections were observed in colonies derived from CC cells grown under the same conditions. As with overall morphology, Δ*miR-100/miR-125b* colony dynamics and projections were more similar to those observed in CC colonies than CC-CR colonies [Supplementary Movies 1-3].

Quantification of overall spheroid growth revealed that colonies derived from CC-CR cells showed a significant increase in total spheroid area compared to colonies derived from CC or Δ*miR-100/miR-125b* cells [Figure 5B]. We also quantified the number and size of projections into the collagen as a measure of invasiveness by determining the increase in projection surface area after growth in collagen for five days. CC-CR colonies showed a significantly greater increase in projection area compared to CC and Δ*miR-100/miR-125b* colonies [Figure 5C]. While invasiveness is likely due to multiple changes, the decreased projections we observe in CC and Δ*miR-100/miR-125b* colonies are consistent with increased expression of CGN due to lower levels of *miR-100* and *miR-125b*.

### *miR-100* and *miR-125b* contribute to enhanced 3D growth and invasiveness in glioblastoma cells

**Figure 6:**
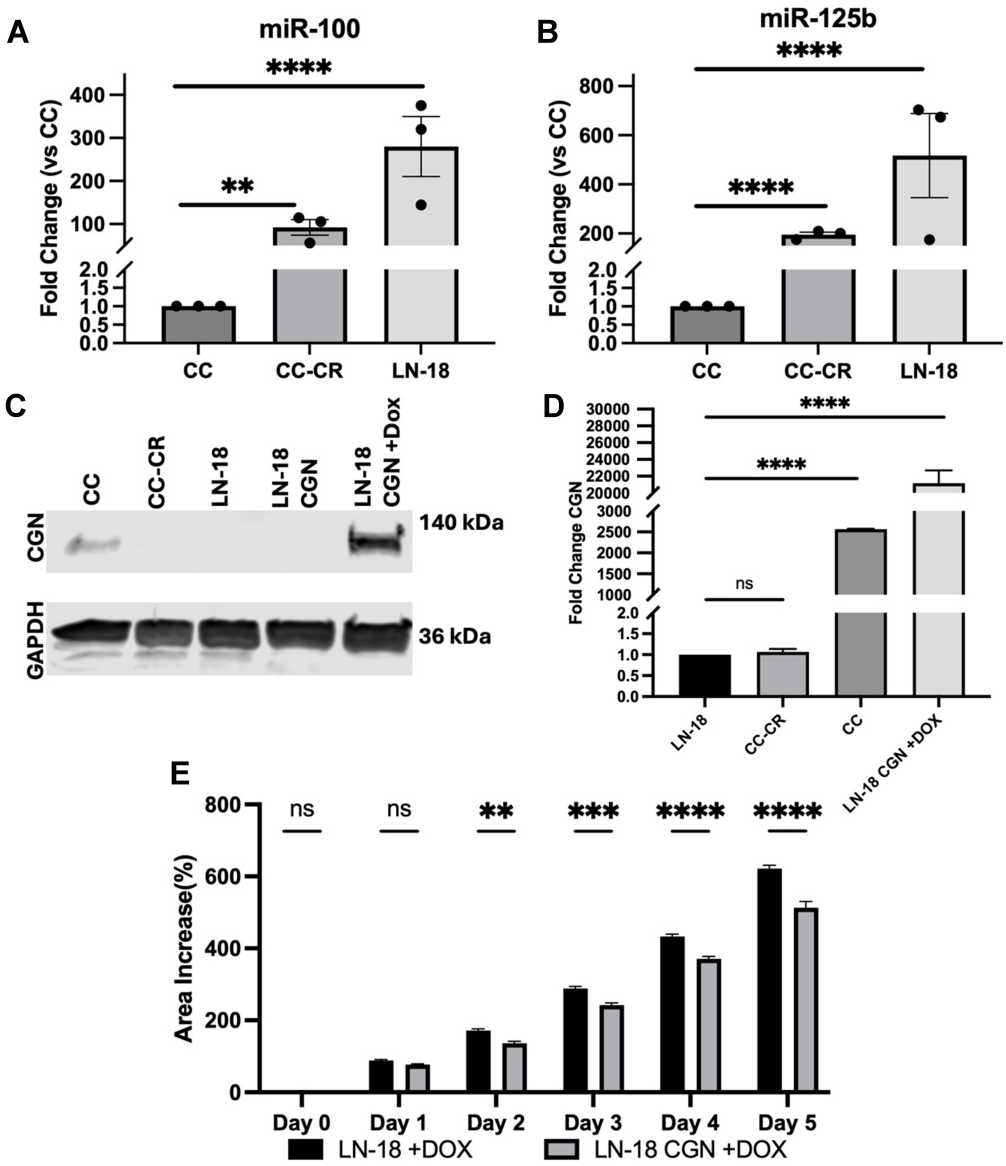
Increased CGN expression in glioblastoma contributes to decreased 3D growth. (A and B) Relative levels of *miR-100* and *miR-125b* in CC, CC-CR, and LN-18 (glioblastoma) cell lines. Data represent mean ± SEM with ^**^ indicating *P* < 0.01 and ^****^ indicating *P* < 0.0001. Significance was determined using Student’s *t* test; *n* = 3; (C) CGN levels were determined using western blots in CC, CC-CR, LN-18, and doxycyclin inducible LN-18 cell lines. Lane loading was monitored with blots against GAPDH; (D) Quantification of blots as in (C) showing fold changes in CGN in the indicated cell lines normalized to GAPDH. Data represent mean ± SEM with *n* = 3. Significance was determined using Student’s *t* test with ^****^ indicating *P* < 0.0001; ns indicating not significant; (E) Increase in spheroid area was measured after transfer to collagen from 0-5 days. Surface area tracing was performed using ImageJ. Significance was determined using two-way ANOVA tests, *n* = 6. Data represent mean ± SEM with ^**^ indicating *P* < 0.01, ^***^ indicating *P* < 0.001, ^****^ indicating *P* < 0.0001; ns indicating not significant. CGN: Cingulin; CC: cystic colonies; SEM: scanning electron microscopy; GAPDH: glyceraldehyde 3-phosphate dehydrogenase; ANOVA: analysis of variance.

To test whether targeting of CGN by *miR-100* and *miR-125b* can alter the growth characteristics of non-CRC cells, we used the LN-18 glioblastoma line^[45]^. To our surprise, these cells express even higher levels of *miR-100* and *miR-125b* than CC-CR cells, consistent with undetectable levels of CGN [Figure 6A-D], consistent with the GENT2 analysis in Supplementary Figure 3A which shows decreased expression in brain cancer. Consistent with our data in LN-18 cells, CGN is not highly expressed in glioma cells within The Cancer Genome Atlas (TCGA). When we plated LN-18 cells under spheroid conditions and transferred the colonies to growth in 3D type I collagen over 5 days, we observed an increase in colony growth [Figure 6E]. However, when we overexpressed CGN levels in LN-18 cells, we detected a significant decrease in colony growth [Figure 6E]. This demonstrates that CGN levels can affect colony growth and that the effect of CGN is not restricted to CRC cells.

### Extracellular transfer of *miR-100* and *miR-125b*

**Figure 7:**
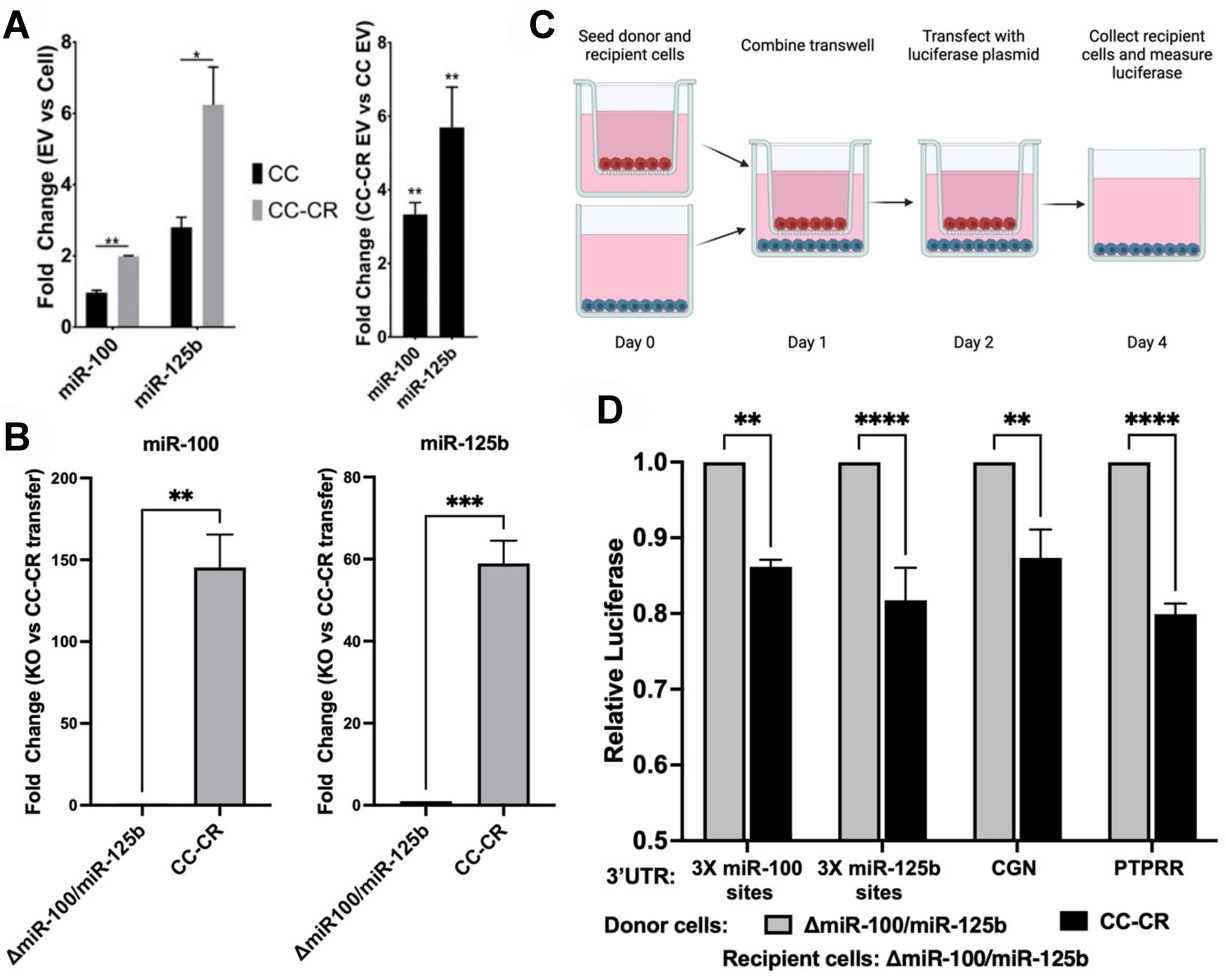
Functional transfer of *miR-100* and *miR-125b*. (A) RNA was collected from EVs from CC and CC-CR cells and the levels of *miR-100* and *miR-125b* were quantified by RT-qPCR. The left panel shows the fold-change enrichment of these miRNAs in EVs compared to cellular levels. The right panel shows the fold change in EV levels of *miR-100* and *miR-125b* when comparing CC-CR and CC cells; (B) Transwell co-culture experiments were performed with Δ*miR-100/miR-125b* recipient cells and the donor cells as indicated on the X-axis. RNA was collected from the recipient cells after 5 days in culture and fold changes in *miR-100* and *miR-125b* levels were determined by RT-qPCR; (C) Schematic of Transwell co-culture experiments to test silencing of CGN and PTPRR luciferase reporter constructs in *miR-100/miR125b* recipient cells; (D) Δ*miR-100/miR-125b* recipient cells were transfected with luciferase reporters and co-cultured with either Δ*miR-100/miR-125b* (grey) or CC-CR (black) donor cells. Relative luciferase values (y-axis) were calculated in recipient cells, normalizing as in Figure 3. For (A and B), data (mean ± SEM, *n* = 3) were analyzed using one-way ANOVA tests while the data in panel (D) (mean ± SEM, *n* = 3) were analyzed using two-way ANOVA tests. For significance, ^*^ indicates *P* < 0.05, ^**^ indicates *P* < 0.01, ^***^ indicates *P* < 0.001, and ^****^ indicates *P* < 0.0001; ns is not significant. EVs: Extracellular vesicles; CC: cystic colonies; RT-qPCR: reverse transcription-quantitative polymerase chain reaction; CGN: cingulin; PTPRR: protein tyrosine phosphatase receptor type-R; SEM: scanning electron microscopy; ANOVA: analysis of variance.

Previous work identified CGN and other targets for *miR-100* and *miR-125b* and showed that cellular changes in these targets can drive migration and invasion of CRC cells^[9]^. Here, we sought to extend those experiments to focus on the extracellular transfer of *miR-100* and *miR-125b* because we had previously shown that these miRNAs can be secreted from CRC cells^[4,46]^. However, definitive evidence of extracellular transfer of these miRNAs was lacking because the experiments used recipient cells that express *miR-100* and *miR-125b* which can lead to indirect silencing of reporter constructs and potential false positives^[18]^. Our knockout cell lines provide an ideal experimental system to conclusively test for extracellular transfer of *miR-100* and *miR-125b* between donor and recipient cells. As donor cells, we used CC-CR cells because they not only express high levels of *miR-100* and *miR-125b*, but they also secrete both miRNAs [Figure 7A]. Thus, we set up Transwell assays with CC-CR donor cells and *miR-100/miR-125b* recipient cells and co-cultured the cells for 5 days before isolation of RNA from the recipient cells and analysis of *miR-100* and *miR-125b* levels. Compared to control transfer experiments in which Δ*miR-100/miR-125b* cells were used as both the donor and recipient cells, we observed dramatic increases in the levels of *miR-100* and *miR-125b* in Δ*miR-100/miR-125b* recipient cells (~150- and 60-fold, respectively) when CC-CR cells were used as the donor [Figure 7B].

Knowing that *miR-100* and *miR-125b* can be successfully transferred between cells, we tested whether their transfer from CC-CR cells to Δ*miR-100/miR-125b* recipient cells can result in functional miRNA transfer. For this, we used Transwell co-culture assays in which the Δ*miR-100/miR-125b* recipient cells were transfected with vectors in which the firefly luciferase open reading frame was fused to the 3’-UTRs from either CGN or PTPRR [Figure 7C]. Compared to controls, we observed a significant reduction in luciferase levels when CC-CR cells were the donor cells and Δ*miR-100/miR-125b* were the recipient cells [Figure 7D]. These data demonstrate that *miR-100 and miR-125b* can be functionally transferred between cells to silence reporter mRNAs. To determine whether *miR-100* and *miR-125b* are transferred as part of EVs, we purified crude small EVs (sEVs) from either CC-CR or Δ*miR-100/miR-125b* cells and incubated them directly with Δ*miR-100/miR-125b* cells expressing luciferase fused to the 3’ UTR of CGN [Supplementary Figure 4]. We observed a modest but significant decrease in luciferase levels when sEVs from CC-CR cells were used as compared to sEVs purified from Δ*miR-100/miR-125b* cells. This supports the notion that *miR-100* and *miR-125b* are part of sEVs but also suggests that other particles may be transferring these miRNAs in the Transwell experiments [Figure 7D], including non-vesicular particles such as exomeres and supermeres which are enriched in miRNAs^[47,48]^.

**Figure 8:**
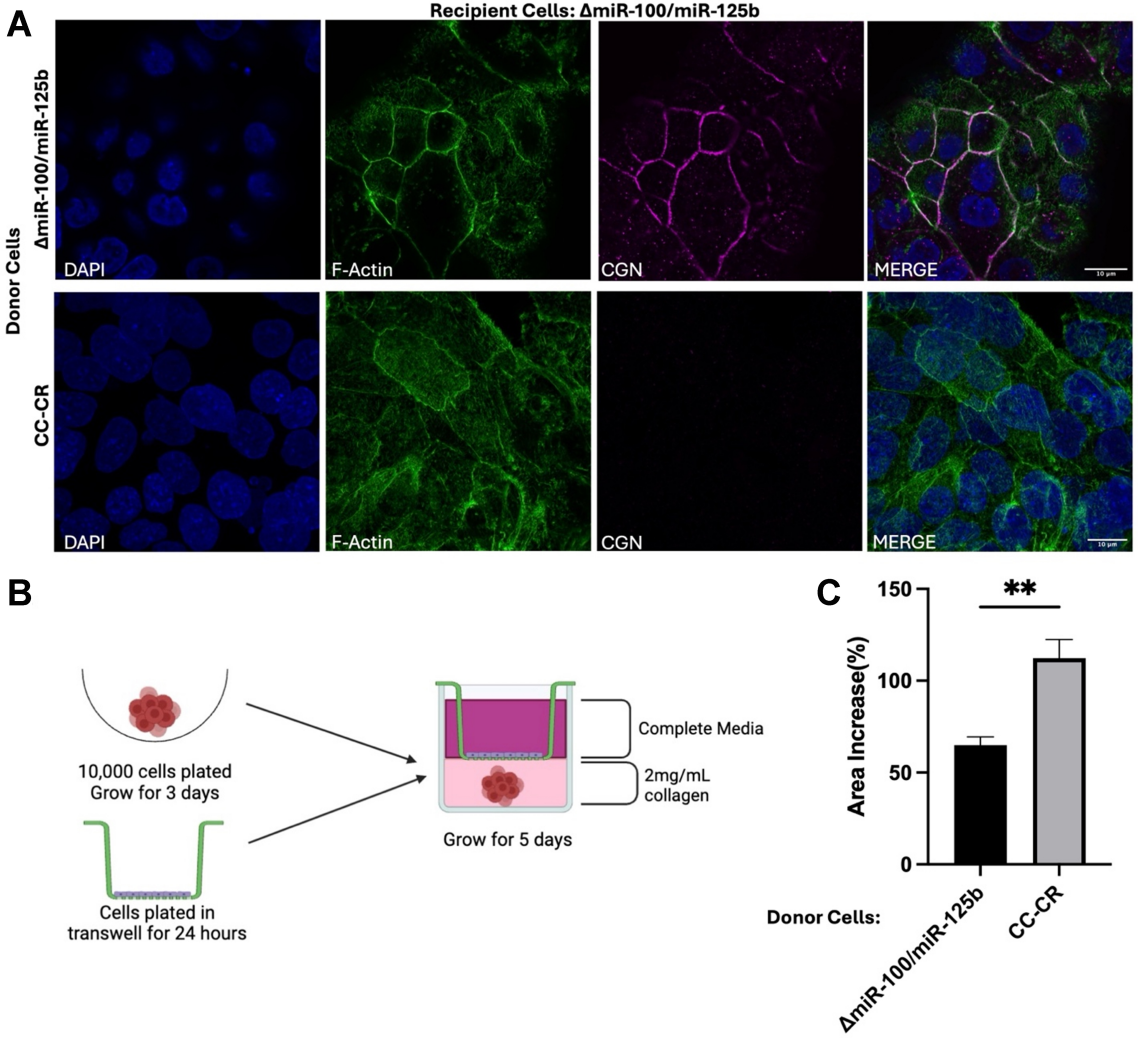
Transfer of *miR-100* and *miR-125b* decreases CGN immunofluorescence in recipient cells. (A) Transwell co-culture experiments were performed with Δ*miR-100/miR-125b* recipient cells and the indicated donor cells. After 5 days, recipient cells were immunostained as in Figure 5C. Scale bar indicates 10 mm; (B) Schematic of Transwell co-culture experiments to test growth changes in spheroids due to transfer of material from donor 2-D cells to recipient spheroids grown in 3D; (C) The increase in spheroid colony area was determined by measuring the surface area of spheroids from day 0 to day 5 after exposure to donor cells. Significance was determined using Student’s *t* test; *n* = 6. Data represent mean ± SEM with ^**^ indicating *P* < 0.01. CGN: Cingulin; SEM: scanning electron microscopy.

To test whether transfer of these miRNAs can silence endogenous CGN in recipient cells, we used Transwell co-culture experiments with Δ*miR-100/miR-125b* recipient cells and either CC-CR or Δ*miR-100/miR-125b* donor cells followed by immunofluorescent staining of CGN in recipient cells. With Δ*miR-100/miR-125b* donor cells, CGN immunostaining was readily apparent in Δ*miR-100/miR-125b* recipient cells [Figure 8A] at levels similar to those observed in Figure 4C. However, when CC-CR cells were used as the donor cells, CGN was undetectable in Δ*miR-100/miR-125b* recipient cells [Figure 8A]. These results demonstrate that endogenous CGN can be targeted by functional transfer of *miR-100* and *miR-125b*, leading to decreased endogenous target protein levels.

As a final assay to test for functional extracellular transfer of *miR-100* and *miR-125b*, we used Transwell experiments in which spheroid cultures were plated in 3D type I collagen and co-cultured with either CC-CR or Δ*miR-100/miR-125b* cells. Exposure of spheroid colonies to CC-CR cells which express high levels of *miR-100* and *miR-125b* led to an increase in the area of the resultant colonies compared to co-culture with Δ*miR-100/miR-125b* cells [Figure 8B and C]. This supports the hypothesis that extracellular transfer of *miR-100* and *miR-125b* can alter 3D growth characteristics and invasiveness, consistent with reduced levels of CGN.

## DISCUSSION

While many reports have suggested that miRNAs can undergo extracellular transfer between cells^[4,49-54]^, the recipient cells in nearly all of these experiments express the same miRNAs, complicating the interpretation of target mRNA silencing after transfer^[18]^. Silencing under these conditions could be due to indirect effects including unexpected increased expression of endogenous miRNAs. Our knockout cell lines provide an ideal experimental system to obviate such concerns. Using donor cells that express high levels of *miR-100* and *miR-125b* and recipient cells with targeted deletions of these genes, we identified mRNA targets and showed functional transfer of *miR-100* and *miR-125b* to silence both reporter constructs and endogenous genes in recipient cells.

All cells release a heterogeneous mixture of membrane-bound vesicles and nanoparticles with current work devoted to understanding biogenesis and cargo loading mechanisms^[16]^. Our experiments conclusively demonstrate extracellular trafficking of *miR-100* and *miR-125b* but whether they are transferred as RNA-protein complexes, nanoparticles (exomeres and supermeres), or membrane-encased small or large vesicles remains to be determined^[47,55]^.

### Tumor microenvironment implications

Invasiveness and metastasis are indicative of differing stages of cancer and can also be indicative of patient prognosis^[56,57]^. EMT is a pivotal stage of metastasis involving the breakdown of cell-cell adhesions^[58]^. Located on apical regions of adjacent epithelial cells, tight junctions play a crucial role in cell-cell adhesion and tissue integrity. Disruption of tight junctions can allow cells to become invasive and metastatic^[59]^. CGN serves as a tether between the cytoskeleton and tight junction components, interacting with ZO-1 at apical junctions^[41,60]^. Loss of CGN in epithelial cells disrupts tight junction formation and cell polarity^[42-44]^. Coupled with our finding that *miR-100* and *miR-125b* are enriched in EVs from CRC cells and that *miR-100* and *miR-125b* can be transferred between cells, our data support a model whereby cell-cell communication within the tumor microenvironment can alter tight junctions, leading to altered growth and increased metastatic potential in recipient cells.

Disruption of tight junctions can also alter the blood-brain barrier in glioblastoma, referred to as the blood-tumor barrier^[61]^. In this case, tight junctions and adherens junctions in endothelial cells are disrupted, leading to impaired barrier function, development of EMT, and increased invasiveness^[62]^. Our finding that glioblastoma cells express high levels of *miR-100* and *miR-125b* and correspondingly low levels of CGN are consistent with the increased invasiveness we observe in 3D colony growth and further support the finding that disruption of tight junctions through decreased CGN levels can facilitate tumor progression through both cell-autonomous and non-cell-autonomous mechanisms.

Our experiments provide a simplified model of cell-cell communication within the tumor microenvironment using Transwell assays with donor and recipient cells. *In vivo*, such communication is extensive, with both tumor and normal (stromal) cells secreting vesicles and nanoparticles that can engage in two-way exchange. The release of EVs and nanoparticles (EVPs) by normal cells, fibroblasts, endothelial cells, and immune cells can inhibit or restrain cancer cell growth, counteracted by the release of EVPs from tumor cells to evade such effects^[63]^. EVPs released by tumor cells can also travel to distant organs and generate the metastatic niche which can remodel local gene expression patterns to enhance metastatic cell growth^[64]^. Both miRNA and protein cargo transfer can mediate this cross-communication, as exemplified by findings that immune modulators such as PD-L1 are carried on tumor EVs that function to suppress immune rejection^[65]^. Beyond cross-communication, it is also possible that the transfer of EVs between tumor cells themselves can enhance the overall progression of events such as EMT by transfer of miRNAs such as *miR-100* and *miR-125b* between cells that express high levels of these miRNAs (and other cargo) compared to lower expressing tumor cells.

### *MIR100HG*, tumor growth, invasiveness and EMT

Beyond demonstrating miRNA transfer between cells, our knockout lines provided a model to test the effects of 3D growth in the presence and absence of *miR-100* and *miR-125b.* In 3D growth in collagen, CC cells form hollow cysts with a central lumen lined by polarized cells, whereas CC-CR cells grow into the central lumen with disorganized, solid colonies^[8]^. A prior deletion of exon 4 within the *MIR100HG* locus supported the idea that *MIR100HG* contributes to both cetuximab resistance and metastasis^[7]^. Our precise deletions of *miR-100* and *miR-125b* allowed us to test the effect of these miRNAs on 3D growth and showed that the loss of these miRNAs results in slower overall growth with decreased edge dynamics and fewer invasive projections. Since the expression of *MIR100HG* is unaffected in our knockout lines, our data agree with previous results that *MIR100HG* can induce EMT, but also that *miR-100* and *miR-125b* play a similar role through the downregulation of CGN^[9]^. The data are also consistent with our GO analysis of the targets of *miR-100* and *miR-125b* being enriched in cell motility and migration [Figure 2C].

In summary, our data support findings that increased expression of *miR-100* and *miR-125b* in cancer promotes disruption of tight junctions, activation of EMT, and increased metastatic potential. Given that these miRNAs are abundant in EVs secreted from CRC and glioblastoma cells, the data support both cell-autonomous and non-cell-autonomous roles for *miR-100* and *miR-125b.* Given that miRNAs are abundant in both vesicular and non-vesicular extracellular particles, a limitation of our studies is that we have not yet defined which particle(s) is most efficient at transferring *miR-100* and *miR-125b* and we have not defined the precise mechanisms as to how these particles are taken up by recipient cells.

## DECLARATIONS

### Availability of data and materials

All RNAseq data are freely available on the GEO website (GSE259371, GSE259370, GSE259373, and GSE259374). All constructs and cell lines are available upon request.

### Copyright

© The Author(s) 2024.
