# Supplementary material for: Transfer of miR-100 and miR-125b increases 3D growth and invasiveness in recipient cancer cells
Source: Extracell Vesicles Circ Nucl Acids. 2024 Jul 29;5(3):397–416. doi: 10.20517/evcna.2024.43 (PMC11648436; doi:10.20517/evcna.2024.43)
Supplement: Supplementary file 1 [file evcna-5-3-397-SupplementaryMaterials.zip › evcna-5-3-397-SupplementaryMaterials/evcna-5-3-397-Supplementary Figures.pdf]

Supplementary Figures

Transfer of *miR-100* and *miR-125b* increases 3D growth and invasiveness in recipient cancer cells

Hannah M. Nelson[1], Shimian Qu[1], Liyu Huang[1], Muhammad Shameer[1], Kevin C. Corn[2], Sydney N. Chapman[1], Nicole L. Luthcke[1], Sara A. Schuster[1], Tellie D. Stamaris[1], Lauren A. Turnbull[1], Lucas L. Guy[1], Xiao Liu[3], Danielle L. Mitchell[4], Elizabeth M. Semler[4], Kasey C. Vickers[4], Qi Liu[3], Jeffrey L. Franklin[5], Alissa M. Weaver[6], Marjan Rafat[2], Robert J. Coffey[7], James G. Patton[1]

[1]Laboratory of James G. Patton, Department of Biological Sciences, Vanderbilt University, Nashville, TN 37235, USA.
[2]Laboratory of Marjan Rafat, Department of Biomedical Engineering, Vanderbilt University, Nashville, TN 37232, USA.
[3]Laboratory of Qi Liu, Department of Biostatistics, Vanderbilt University Medical Center, Nashville, TN 37232, USA.
[4]Laboratory of Kasey C. Vickers, Department of Molecular Physiology and Biophysics, Vanderbilt University Medical Center, Nashville, TN 37232, USA.
[5]Department of Cell and Developmental Biology, Vanderbilt University Medical Center, Nashville, TN 37235, USA.
[6]Laboratory of Alissa M. Weaver, Department of Cell and Developmental Biology, Vanderbilt University Medical Center, Nashville, TN 37235, USA.
[7]Laboratory of Robert J. Coffey, Department of Medicine, Division of Gastroenterology, Hepatology and Nutrition, Vanderbilt University Medical Center, Nashville, TN 37232, USA.

Correspondence to: Dr. James G. Patton, Laboratory of James G. Patton, Department of Biological Sciences, Vanderbilt University, Nashville, TN 37235, USA. E-mail: james.g.patton@vanderbilt.edu

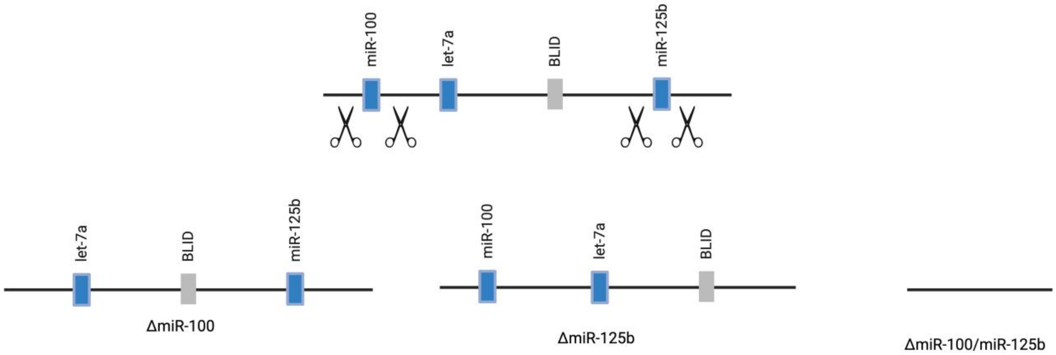

Supplementary Figure 1. Generation of Δ*miR-100*, Δ*miR-125b*, and Δ*miR-100/miR-125b* CC-CR cells. gRNAs complementary to sequences flanking *miR-100*, *miR-125b*, or both (gRNAs on the far left and right), were used to direct CRISPR/Cas9 (scissors) to generate deletions within the MIR100HG locus in CC-CR cells.

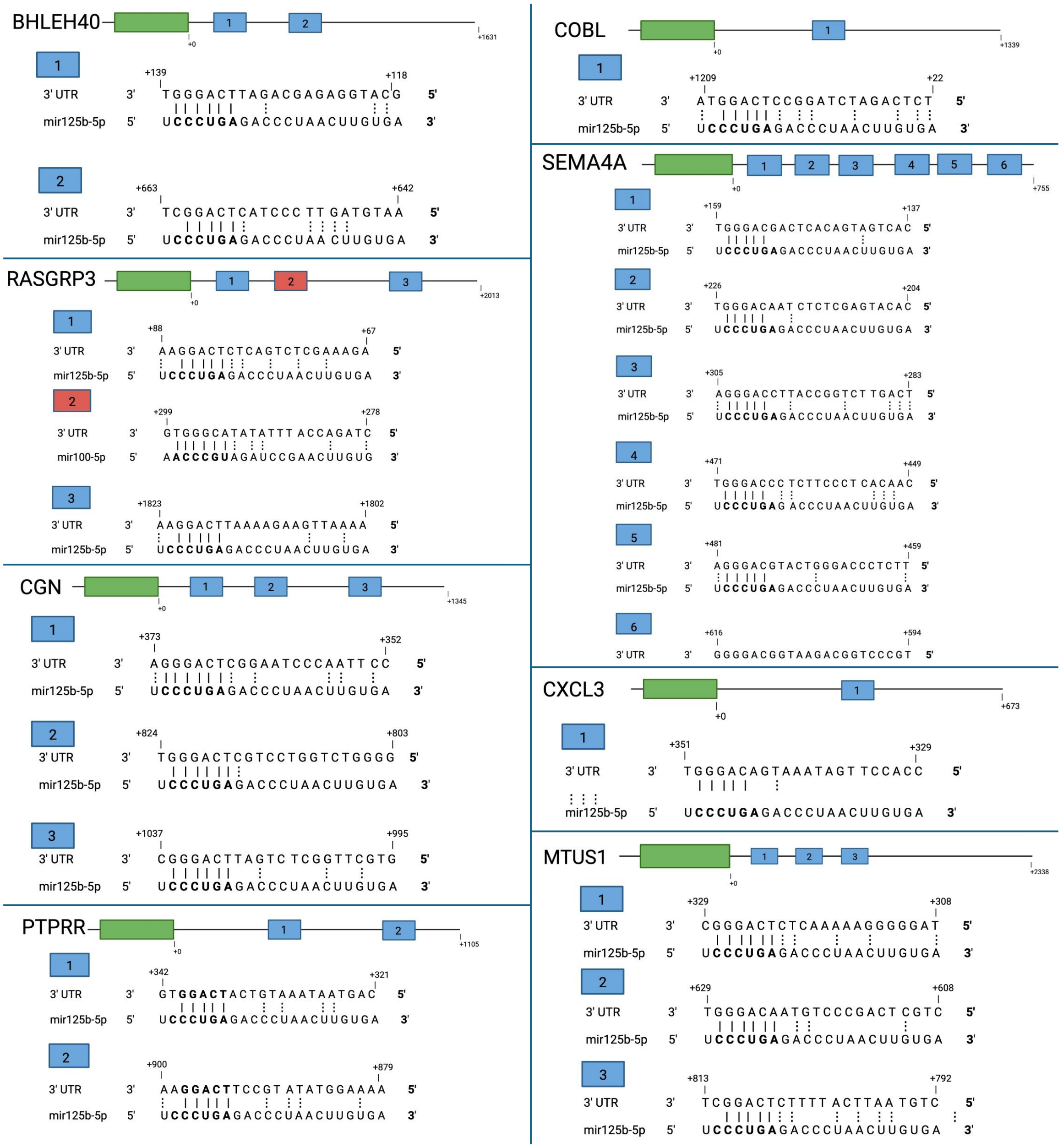

Supplementary Figure 2. Pairing between *miR-100* and *miR-125b* and candidate targets. Pairing between the 8 statistically significant candidate targets verified by luciferase assay are shown. Each mRNA is shown with the coding region in green followed by the 3'-UTR. Binding sites for *miR-100* (red) and *miR-125b* (blue) are as indicated. Solid lines indicate potential pairing between the mRNA and the seed sequence at the 5' end of the each miRNA. Dotted lines indicate potential base pairing outside of the seed sequence.

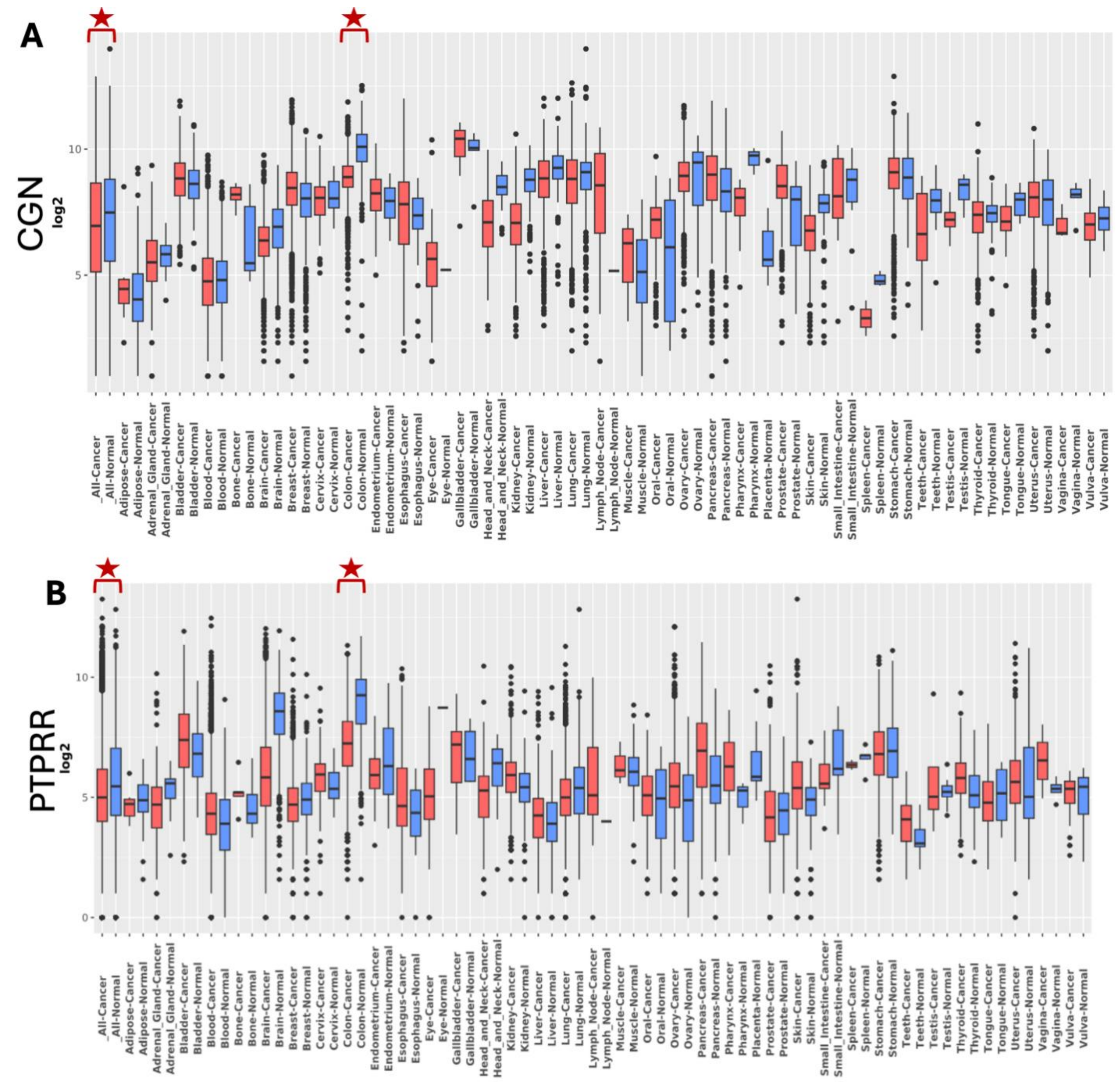

Supplementary Figure 3. Decreased Expression of CGN and PTPRR in cancer. Boxplot of CGN (A) and PTPRR (B) expression across 72 paired tissues. Red indicates cancer samples, blue indicates normal samples. Stars and red brackets highlight comparisons between normal tissues and all cancers or between normal tissues and colon cancer.

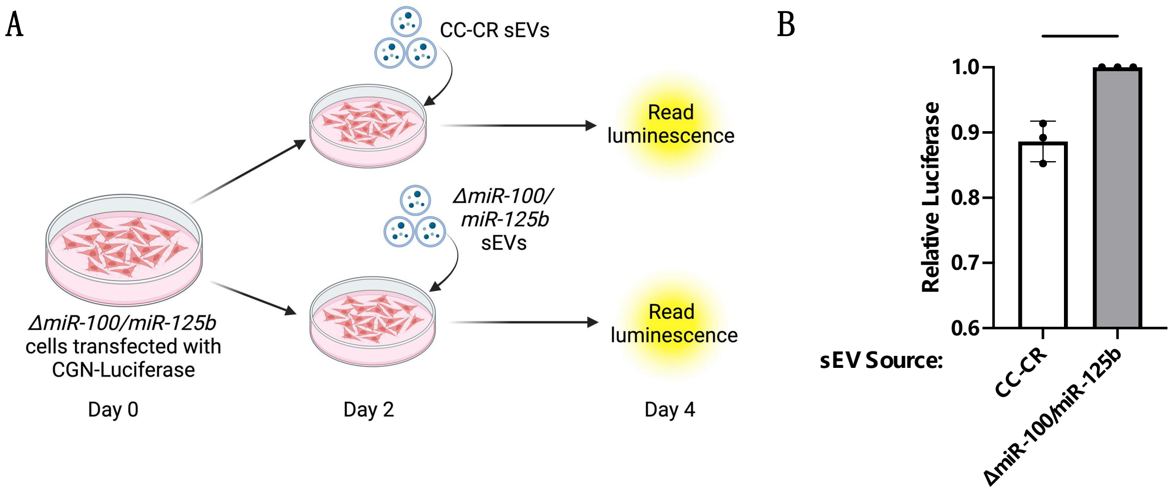

Supplementary Figure 4. Transfer of *miR-100* and *miR-125b* in sEVs. (A) Schematic or experimental design. (B) Small EVs (sEVs) were isolated from either CC-CR cells or Δ*miR-100/miR-125b* cells and incubated with *miR-100 and miR-125b* cells expressing luciferase with the its open reading frame fused to the Cingulin 3' UTR. The modest but significant decrease in luciferase levels when using sEVs from CC-CR cells indicates transfer of *miR-100* and *miR-125b*.
