# Supplementary material for: Transfer of miR-100 and miR-125b increases 3D growth and invasiveness in recipient cancer cells
Source: Extracell Vesicles Circ Nucl Acids. 2024 Jul 29;5(3):397–416. doi: 10.20517/evcna.2024.43 (PMC11648436; doi:10.20517/evcna.2024.43)
Supplement: Supplementary file 1 [file evcna-5-3-397-SupplementaryMaterials.zip › evcna-5-3-397-SupplementaryMaterials/evcna-5-3-397-Supplementary Tables.pdf]

Supplementary Tables

Transfer of *miR-100* and *miR-125b* increases 3D growth and invasiveness in recipient cancer cells

Hannah M. Nelson[1], Shimian Qu[1], Liyu Huang[1], Muhammad Shameer[1], Kevin C. Corn[2], Sydney N. Chapman[1], Nicole L. Luthcke[1], Sara A. Schuster[1], Tellie D. Stamaris[1], Lauren A. Turnbull[1], Lucas L. Guy[1], Xiao Liu[3], Danielle L. Mitchell[4], Elizabeth M. Semler[4], Kasey C. Vickers[4], Qi Liu[3], Jeffrey L. Franklin[5], Alissa M. Weaver[6], Marjan Rafat[2], Robert J. Coffey[7], James G. Patton[1]

[1]Laboratory of James G. Patton, Department of Biological Sciences, Vanderbilt University, Nashville, TN 37235, USA.

[2]Laboratory of Marjan Rafat, Department of Biomedical Engineering, Vanderbilt University, Nashville, TN 37232, USA.

[3]Laboratory of Qi Liu, Department of Biostatistics, Vanderbilt University Medical Center, Nashville, TN 37232, USA.

[4]Laboratory of Kasey C. Vickers, Department of Molecular Physiology and Biophysics, Vanderbilt University Medical Center, Nashville, TN 37232, USA.

[5]Department of Cell and Developmental Biology, Vanderbilt University Medical Center, Nashville, TN 37235, USA.

[6]Laboratory of Alissa M. Weaver, Department of Cell and Developmental Biology, Vanderbilt University Medical Center, Nashville, TN 37235, USA.

[7]Laboratory of Robert J. Coffey, Department of Medicine, Division of Gastroenterology, Hepatology and Nutrition, Vanderbilt University Medical Center, Nashville, TN 37232, USA.

Correspondence to: Dr. James G. Patton, Laboratory of James G. Patton, Department of Biological Sciences, Vanderbilt University, Nashville, TN 37235, USA. E-mail: james.g.patton@vanderbilt.edu

Supplementary Table 1. Oligonucleotide primers

Table showing construct names and the forward (F) and reverse (R) primers used for PCR amplification or cloning of 3' elements inserted into luciferase expression vectors. These include genes as listed by their abbreviations or sequences containing perfect binding sites for *miR-100* or *miR-125b*. Perfect binding sites for *miR-100* and *miR-125b* were generated by annealing the indicated forward and reverse oligonucleotides. Guide RNA (gRNA) sequences for CRISPR/Cas9 mediated cleavage of the MIR100HG locus are as shown.

Supplementary Table 2. Long RNAseq data

RNA (> 200 nt) was isolated and RNAseq was performed on CC-CR, Δ*miR-100*, Δ*miR-125b*, and Δ*miR-100/miR-125b* cells. Differentially expressed genes with absolute fold change > 2 or < 0.5, and adjusted *P* values (padj) < 0.05 were determined using DESeq2 (v1.30.1) (Love *et al.*, 2014). RNAseq data and differential expression analyses for the different comparisons are represented by different spreadsheets under each tab.

Supplementary Table 3. Small RNAseq data from AGO2 RNA immunoprecipitation from CC and CC-CR cells

RNA sequencing data for libraries generated from RNA < 200 nt isolated CC and CC-CR cells, as well as RNA isolated after AGO2 RNA immunoprecipitation. Small RNAseq data sets are represented by different tabs within the larger file. Significant differentially expressed genes with absolute fold change > 2 or < 0.5, and adjusted *P* values (padj) < 0.05 were determined using DESeq2 (v1.30.1) (Love *et al.*, 2014).

Supplementary Table 4. Long RNAseq data from AGO2 RNA immunoprecipitation from CC and CC-CR cells

RNA sequencing data for libraries generated from RNA > 200 nt isolated from CC and CC-CR cells, as well as RNA isolated after AGO2 RNA immunoprecipitation. Long RNAseq data sets are represented by different tabs within the larger file. Significant differentially expressed genes with absolute fold change > 2 or < 0.5, and adjusted *P* values (padj) < 0.05 were determined using DESeq2 (v1.30.1) (Love *et al.*, 2014).

Supplementary Table 5. Gene ontology analysis of targets of *miR-100* and *miR-125b*

Gene ontology analyses were performed examining enriched genes among all 96 candidate targets for both *miR-100* and *miR-125b*, for each miRNA alone, and for the 15 candidates that were directly tested using luciferase assays.
