# Supplementary material for: Transfer of miR-100 and miR-125b increases 3D growth and invasiveness in recipient cancer cells
Source: Extracell Vesicles Circ Nucl Acids. 2024 Jul 29;5(3):397–416. doi: 10.20517/evcna.2024.43 (PMC11648436; doi:10.20517/evcna.2024.43)
Supplement: Supplementary file 1 [file evcna-5-3-397-SupplementaryMaterials.zip › evcna-5-3-397-SupplementaryMaterials/evcna-5-3-397-upplementary Movies.pdf]

Supplementary Movies

Transfer of *miR-100* and *miR-125b* increases 3D growth and invasiveness in recipient cancer cells

Hannah M. Nelson[1], Shimian Qu[1], Liyu Huang[1], Muhammad Shameer[1], Kevin C. Corn[2], Sydney N. Chapman[1], Nicole L. Luthcke[1], Sara A. Schuster[1], Tellie D. Stamaris[1], Lauren A. Turnbull[1], Lucas L. Guy[1], Xiao Liu[3], Danielle L. Mitchell[4], Elizabeth M. Semler[4], Kasey C. Vickers[4], Qi Liu[3], Jeffrey L. Franklin[5], Alissa M. Weaver[6], Marjan Rafat[2], Robert J. Coffey[7], James G. Patton[1]

[1]Laboratory of James G. Patton, Department of Biological Sciences, Vanderbilt University, Nashville, TN 37235, USA.

[2]Laboratory of Marjan Rafat, Department of Biomedical Engineering, Vanderbilt University, Nashville, TN 37232, USA.

[3]Laboratory of Qi Liu, Department of Biostatistics, Vanderbilt University Medical Center, Nashville, TN 37232, USA.

[4]Laboratory of Kasey C. Vickers, Department of Molecular Physiology and Biophysics, Vanderbilt University Medical Center, Nashville, TN 37232, USA.

[5]Department of Cell and Developmental Biology, Vanderbilt University Medical Center, Nashville, TN 37235, USA.

[6]Laboratory of Alissa M. Weaver, Department of Cell and Developmental Biology, Vanderbilt University Medical Center, Nashville, TN 37235, USA.

[7]Laboratory of Robert J. Coffey, Department of Medicine, Division of Gastroenterology, Hepatology and Nutrition, Vanderbilt University Medical Center, Nashville, TN 37232, USA.

Correspondence to: Dr. James G. Patton, Laboratory of James G. Patton, Department of Biological Sciences, Vanderbilt University, Nashville, TN 37235, USA. E-mail: james.g.patton@vanderbilt.edu

Movie available on BioRxiv under Supplemental Movie 1
https://www.biorxiv.org/content/10.1101/2024.01.16.575716v1.supplementary-material

Supplementary Movie 1. Timelapse movie of Δ*miR-100/miR-125b* spheroids. Δ*miR-100/miR-125b* spheroids were grown in collagen with images taken every hour for five days. Movie (18 sec) consists of images taken every hour over 5 days.

Movie available on BioRxiv under Supplemental Movie 2
https://www.biorxiv.org/content/10.1101/2024.01.16.575716v1.supplementary-material

Supplementary Movie 2. Timelapse movie of CC spheroids. CC spheroids were grown in collagen with images taken every hour for five days. Movie (18 sec) consists of images taken every hour over 5 days.

Movie available on BioRxiv under Supplemental Movie 3
https://www.biorxiv.org/content/10.1101/2024.01.16.575716v1.supplementary-material

Supplementary Movie 3. Timelapse movie of CC-CR spheroids. CC-CR spheroids were grown in collagen with images taken every hour for five days. Movie (18 sec) consists of images taken every hour over 5 days.
